# Clinical characteristics and outcomes of patients who underwent neonatal cardiac surgery: ten years of experience in a tertiary surgery center

**DOI:** 10.1186/s40001-024-01735-5

**Published:** 2024-02-26

**Authors:** Han Zhang, Gang Li, Qiangqiang Li, Yansong Zuo, Qiang Wang

**Affiliations:** Beijing Anzhen Hospital, Capital Medical University, 2 Anding Road, Beijing, 100029 China

**Keywords:** Neonatal cardiac surgery, Survival, Outcome, Risk

## Abstract

**Objective:**

To evaluate the outcomes after neonatal cardiac surgery at our institute, and identify factors associated with operative mortality.

**Methods:**

We examined 224 neonates who underwent cardiac surgery at a single institution from 2013 to 2022. Relevant data, such as demographic information, operative details, and postoperative records, were gathered from medical and surgical records. Our primary focus was on the operative mortality.

**Results:**

Median age and weight at surgery were 12 (7–20) days and 3.4 (3.0–3.8) kg, respectively. Overall mortality was 14.3% (32/224). Mortality rates showed improvement over time (2013–2017 vs. 2018–2022), with rates decreasing from 21.9% to 10.6% (*p* = 0.023). ECMO use, extubation failure, lactate > 4.8 mmol/l and VIS > 15.5 on 24 h after operation were significantly associated with operative mortality, according to multivariate logistic regression analysis. Patients admitted to the cardiac intensive care unit (CICU) before surgery and those with prenatal diagnosis showed lower operative mortality. Median follow-up time of 192 hospital survivors was 28.0 (11.0–62.3) months. 10 patients experienced late deaths, and 7 patients required reinterventions after neonatal cardiac surgery. Risk factors for composite end-point of death and reintervention on multivariable analysis were: surgical period (HR = 0.230, 95% CI 0.081–0.654; *p* = 0.006), prolonged ventilation (HR = 4.792, 95% CI 1.296–16.177; *p* = 0.018) and STAT categories 3–5 (HR = 5.936, 95% CI 1.672–21.069; *p* = 0.006).

**Conclusions:**

Our institution has observed improved surgical outcomes in neonatal cardiac surgery over the past five years with low mortality, but late death and reintervention remain necessary in some patients. The location and prenatal diagnosis prior to surgery may affect the outcomes of neonates undergoing congenital heart disease operations.

## Introduction

Congenital heart disease (CHD) is now the most common birth defect in China and affects 8.9% of live births [1]. Although advancements in pediatric cardiac surgery, anesthesia, extracorporeal circulation, ultrasound, and intensive care technology have significantly improved the survival rates in neonates, significant morbidity still remains [2]. Risk models currently incorporate factors such as age, weight, preoperative status, comorbidities and surgical complexity, which are associated with increased morbidity and mortality [3]. When assessing early procedural outcomes among centers, it is important to take into account the unique circumstances of each patient. We conducted a study to determine the early outcomes of neonatal congenital heart disease treatments at our institution and to identify risk factors associated with adverse outcomes during long-term follow-up.

## Materials and methods

### Study population

A retrospective cohort study at Beijing Anzhen Hospital examined cardiac surgeries for neonates born from Jan 2013 to Dec 2022. Only patients under 28 days old at the time of operation were included. The study received approval from our institutional Research Ethics Board.

Patient information was obtained retrospectively from medical records with only the first intervention analyzed. Demographic and perioperative data were collected, including age, weight, gestational age (GA), sex, cardiac diagnosis, prenatal diagnosis, admission location, emergent procedure, and preoperative medical data. Surgical procedure complexity was assessed using The Society of Thoracic Surgeons–European Association for Cardio-thoracic Surgery Congenital Heart Surgery (STAT) mortality scores. The data collected during surgery included details about the procedure performed, the amount of time spent on cardiopulmonary bypass, the duration of aortic cross clamp, blood lactate levels, and whether circulatory arrest was needed. After surgery, the data collected included how long mechanical ventilation was required, the vasoactive-inotropic score, blood lactate levels, length of stay (LOS) in the ICU, length of stay in hospital**,** delayed sternal closure, unplanned reoperation, extracorporeal membrane oxygenation (ECMO) use, extubation failure, residual lesion, and whether there were any major postoperative complications such as arrhythmia requiring therapy, complete heart block requiring a pacemaker, diaphragm paralysis, chylothorax, paralyzed diaphragm, acute kidney injury, hepatic injury, wound infection, stroke, pleural effusion requiring drainage, bloodstream infection, or necrotizing enterocolitis. The early outcome was all-cause mortality before discharge from hospital.

### Definitions

Definitions for the study included operative mortality (death prior to hospital discharge), prematurity (birth before 37 weeks gestation), hepatic injury (aspartate aminotransferase (AST) or alanine aminotransferase (ALT) levels greater than 2 times the upper limit of normal) [4], renal injury (an increase in creatinine greater than 2 times the upper limit of normal) [5], prolonged mechanical ventilation (duration exceeding the 75th percentile of mechanical ventilation duration after surgery), extubation failure (unplanned reintubation within 72 h of planned first extubation), residual lesion (residual shunt or obstruction, moderate or severe regurgitation), unplanned reoperation (reoperation during the postoperative period, except for bleeding), arrhythmia requiring therapy (any arrhythmia needing drugs, electrical cardioversion, or defibrillation), bloodstream infection (postoperative positive blood culture result). A vasoactive-inotropic score (VIS) is used to quantify the amount of inotropic support provided in the postoperative period. For each patient, the maximum VIS was recorded during the first 24 postoperative hours, and the VIS was calculated as follows: dopamine dose (μg/kg/min) + dobutamine dose (μg/kg/min) + 100 × epinephrine dose (μg/kg/min)] + 10 × milrinone dose (μg/kg/min) + 10,000 × vasopressin dose (units/kg/min) + 100 × norepinephrine dose (μg/kg/min)) [6]. And routine blood lactate levels were collected at intervals during the postoperative period.

Patients were followed up by clinic visits and telephone contacts. The date of primary operation was considered as baseline for the survival analysis. We defined the composite end-point as all-cause mortality and reintervention after discharged from hospital.

### Statistical analysis

The study utilized the Statistical Package for Social Sciences (SPSS) 25.0 software (SPSS Inc, Chicago, IL, USA) and GraphPad Prism 6 (GraphPad Software, Inc, La Jolla, CA, USA) for statistical analysis. Demographics, patient characteristics, and outcomes were expressed as median (interquartile range) or frequency (%), depending on the variable type. Categorical variables were tested using χ^2^, while numeric variables were evaluated using Mann–Whitney *U* tests. Non-parametric data were assessed using the Kruskal–Wallis test for more than two groups. Univariate and multivariate logistic regression analyses were conducted to determine significant predictors of mortality and prolonged mechanical ventilation, presented as OR and 95%CI. The predictive accuracy of lactate and VIS was explored using receiver operating characteristic (ROC) curves and relative area under the curve (AUC). Different cutoff points were examined for sensitivity and specificity. Survival analysis was performed by Kaplan–Meier curves and log-rank test. Multivariate models were applied using Cox proportional hazards for outcomes. Mortality trends over two time frames (2013–2017 and 2018–2022) were analyzed. A significance level of *P* < 0.05 was considered for all statistical analyses.

## Results

### Patient characteristics

Of the 250 neonates diagnosed, 26 did not receive surgery. A total of 15 patients (57.7%) were diagnosed with TGA/IVS, 3 patients (11.5%) with TAPVC, 1 patient (3.8%) with PA/IVS, 3 patients (11.5%) with IAA and 4 patients (15.4%) with TGA/VSD (Table 1). There were 224 neonates who underwent cardiac surgery; 67.4% (150) were male, and the gestational age was 38 (38–39) weeks. Of these patients, 6.3% (14) had a gestational age < 37 weeks, and the mean age at admission was 5 (0–14) days. Sixty-seven patients were referred from other hospitals, while 157 patients were delivered at our hospital. A prenatal diagnosis of CHD was established in 127 patients. Patients who were delivered in our hospital were more likely to have a prenatal CHD diagnosis (93.0% vs. 34.1%, *p* < 0.05). Eighty-five (37.9%) patients were admitted to the pediatric ward, 119 (53.1%) were admitted to the cardiac intensive care unit (CICU), and 20 (8.9%) were admitted to the neonatal intensive care unit (NICU). Echocardiography revealed that transposition of the great arteries with an intact ventricular septum (TGA/IVS, *n* = 63, 28.1%), total anomalous pulmonary venous connection (TAPVC, *n* = 52, 23.3%), and pulmonary atresia (PA, *n* = 32, 14.3%) were the most common cardiac malformations (Table 2). The STAT mortality scores were used to evaluate the complexity of the operations.

**Table 1:** Characteristics of the patients who did not receive surgery

	Survival group (*n* = 20)	Mortality group (*n* = 6)	*P* value
2013–2017, *n* (%)	12 (70.6)	5 (29.4)	
2018–2022, *n* (%)	8 (88.9)	1 (11.1)	
Age at admission, days	6 (0–15.5)	9.5 (0–19.0)	0.929
Weight, kg	3.1 (2.8–3.5)	3.5 (2.8–4.2)	0.387
Male, *n* (%)	13 (65.0)	5 (83.3)	0.393
Gestational age, weeks	38 (37–39)	38 (37–39)	0.700
< 37 wk *n* (%)	3 (15.0)	0 (0.0)	
Prenatal diagnosis of CHD, *n* (%)	8 (40.0)	2 (33.3)	0.768
Preoperative location, *n* (%)			0.227
Pediatric ward	9 (45.0)	5 (83.3)	
NICU	4 (20.0)	0 (0)	
CICU	7 (35.0)	1 (16.7)	
Preoperative emergent resuscitation, *n* (%)	1 (5.0)	5 (83.3)	0.000
Preoperative respiratory support, *n* (%)			0.001
Noninvasive ventilation	17 (85.0)	1 (16.7)	
Invasive mechanical ventilation	3 (15.0)	5 (83.3)	
Diagnose, *n* (%)			0.125
TGA/IVS	11 (55.0)	4 (66.7)	
TGA/VSD	2 (10.0)	2 (33.3)	
TAPVC	3 (15.0)	0 (0.0)	
IAA	3 (15.0)	0 (0.0)	
PA/IVS	1 (5.0)	0 (0.0)	
Preoperative lac, mmol/L	2.0 (1.5–2.9)	6.5 (4.5–11.9)	0.016

*TGA/IVS* transposition of the great arteries with an intact ventricular septum, *TGA/VSD* transposition of the great arteries with ventricular septal defect, *TAPVC* total anomalous pulmonary venous connection, *IAA* interruption of the aortic arch, *PA/IVS* pulmonary atresia with an intact ventricular septum

**Table 2:** Cardiac diagnosis, operational style and in-hospital mortality rate

	Total (*n*, %)	Operational style (mortality, %)		
		Radical operation	BT	BT + banding
TGA/IVS	63, 28.1%	5.4%	–	1.3%
TGA/VSD	14, 6.3%	–	–	–
TAPVC	52, 23.3%	2.2%	–	–
PA/IVS	28,12.5%	1.8%	0.4%	–
PA/VSD	4,1.9%	–	–	–
PS	17, 7.6%	0.8%	–	–
COA	16, 7.1%	0.4%	–	–
IAA	10, 4.5%	1.3%	–	–
VSD	8, 3.6%	–	–	–
TOF	6, 2.7%	–	–	–
APW	1, 0.4%	–	–	–
Vascular ring	1, 0.4%	–	–	–
AOPA	1, 0.4%	–	–	–
SAS	1, 0.4%	1	–	–
PDA	1, 0.4%	–	–	–
TA	1, 0.4%	–	–	–

*TGA/IVS* transposition of the great arteries with an intact ventricular septum, *TGA/VSD* transposition of the great arteries with ventricular septal defect, *TAPVC* total anomalous pulmonary venous connection, *PA/IVS* pulmonary atresia with an intact ventricular septum, *PA/VSD* pulmonary atresia with ventricular septal defect, *PS* pulmonary stenosis, *COA* coarctation of aorta, *IAA* interruption of the aortic arch, *VSD* ventricular septal defect, *TOF* tetralogy of Fallot, *APW* aortopulmonary window, *AOPA* anomalous origin of pulmonary artery from the ascending aort, *SAS* supravalvular aortic stenosis, *PDA* patent ductus arteriosus, *TA* truncus arteriosus

### Procedures and outcomes

Six neonates died before they could undergo surgery—4 who were born prematurely. Four patients died due to respiratory failure, and 2 patients died due to sudden cardiac death. In the past five years, there was a decrease in the incidence of preoperative death compared to that in previous periods (11.1% in 2018–2022 vs. 29.4% in 2013–2017; *p* = 0.292). Additionally, more patients received surgical treatment.

Of the 224 patients included in the study, 97.3% received anatomic correction, while 2.7% received palliative pulmonary artery banding and/or a Blalock–Taussig shunt. Cardiac surgery via cardiopulmonary bypass was performed in 90.3% of patients, with a median age of 12 (7–20) days and a body weight of 3.4 (3.0–3.8) kg. Thirty patients required an emergent operation, and 32 patients died before discharge, resulting in an all-cause in-hospital mortality rate of 14.3% (Table 2).

Postoperatively, 9 (4.0%) patients required ECMO support, the indications of ECMO conduction were cardiac arrest for 5 cases, failure to wean from cardiopulmonary bypass for 4 cases. And the overall survival to hospital discharge for ECMO was 22.2% (2/9).

Patients who did not survive had earlier GA at birth, were less likely to be prenatally diagnosed and admitted to the CICU, were more likely to require emergent intervention, and had higher lactate levels before and after corrective surgery. Delayed sternal closure, peritoneal dialysis, residual lesion, extubation failure, arrhythmia requiring therapy, and bloodstream infection were also more common in the mortality group (*p* < 0.05) (Table 3). The significant factors associated with mortality were ECMO use, extubation failure, lactate > 4.8 mmol/l and VIS > 15.5 at 24 h after the operation (Table 4).

**Table 3:** Comparison between survival and mortality groups

	Survival group (*n* = 192)	Mortality group (*n* = 32)	*P* value
Age at surgery, days	12 (6–19)	13 (7–20)	0.187
Weight, kg	3.4 (3.0–3.8)	3.3 (3.0–3.6)	0.546
Male, *n* (%)	129 (67.2)	22 (68.8)	0.861
Gestational age at birth, weeks	38 (38–39)	38 (37–38)	0.006
< 37 wk *n* (%)	12 (6.3)	2 (6.3)	1.000
Prenatal diagnosis of CHD, *n* (%)	114 (59.4)	13 (40.6)	0.006
Preoperative location, *n* (%)			0.019
Pediatric ward	68 (35.4)	17 (53.1)	
NICU	12 (6.3)	8 (25.0)	
CICU	112 (58.3)	7 (21.9)	
Preoperative emergent resuscitation, *n* (%)	26 (13.5)	6 (18.8)	0.436
Emergent procedure, *n* (%)	19 (9.9)	11 (34.3)	0.000
Preoperative vasoactive support, *n* (%)	36 (18.8)	8 (25.0)	0.410
Preoperative respiratory support, *n* (%)			0.074
Room air	134 (69.8)	21 (65.6)	
Noninvasive ventilation	25 (13.0)	1 (3.1)	
Invasive mechanical ventilation	33 (17.2)	10 (31.3)	
STAT, *n* (%)			0.460
1	16 (83.3)	0 (0)	
2	87 (45.3)	17 (53)	
3	18 (9)	2 (6)	
4	70 (36)	13 (41)	
5	1 (0.5)	0 (0)	
Preoperative lac, mmol/L	1.7 (1.0–2.9)	2.1 (1.4–5.2)	0.016
Lactate on ICU arrival, mmol/L	2.8 (1.8–4.5)	7.8 (4.4–14.5)	0.000
Lac on 24 h after operation, mmol/L	2.2 (1.5–3.3)	5.5 (3.5–15.0)	0.000
Bypass, *n* (%)	180 (94)	30 (94)	1.000
Bypass time, minutes	150 (102–193)	196 (90–252)	0.027
Aortic cross‑clamp time, minutes	78 (54–110)	89 (44–123)	0.887
Circulatory arrest	28 (15)	4 (13)	0.873
Postoperative mechanical ventilation, hours	90 (48–140)	107 (69–219)	0.049
Postoperative ICU LOS, days	10 (7–15)	8 (4–12)	0.028
Postoperative hospital LOS, days	15 (11–20)	11 (4–16)	0.001
VIS	10 (8–17.5)	23 (15–30)	0.000
ECMO use	2 (1.0)	7 (21.9)	0.000
Delayed sternal closure	46 (24.0)	21 (65.6)	0.000
Peritoneal dialysis	23 (12.0)	22 (68.8)	0.000
Residual lesion, *n* (%)	12 (6.3)	9 (28.1)	0.000
Extubation failure, *n* (%)	15 (7.8)	8 (25.0)	0.000
Major postoperative complications			
Unplanned reoperation, *n* (%)	18 (9.4)	6 (18.8)	0.112
Arrhythmia requiring therapy	18 (9.4)	9 (28.1)	0.003
Complete heart block required PPM	2 (1.0)	0 (0)	0.562
Chylothorax	2 (1.0)	0 (0)	0.562
Paralyzed diaphragm	1 (0.5)	1 (3.1)	0.147
Acute kidney injury (AKI)	23 (12.0)	3 (9.4)	0.670
Hepatic injury	17 (8.9)	13 (40.6)	0.000
Wound infection	8 (4.2)	2 (6.3)	0.597
Stroke	0 (0)	3 (9.4)	0.000
Pleural effusion requiring drainage	4 (2.1)	5 (15.6)	0.000
Atelectasis	42 (21.9)	7 (21.9)	1.000
Bloodstream infection (sepsis)	25 (13.0)	9 (28.1)	0.027
NEC	1 (0.5)	0 (0)	0.682

**Table 4:** Multivariable analysis of factors associated with operative mortality

Variable	*β*	OR	95% CI	AUC (%)	Cut-off value	*p* value
Lac on 24 h after operation	0.262	1.299	1.120–1.506	0.833	4.8	0.000
VIS	0.066	1.068	1.006–1.134	0.803	15.5	0.031
Extubation failure	1.604	4.972	1.462–16.908			0.010
ECMO use	3.878	48.317	6.644–351.392			0.000

**Fig. 1:**
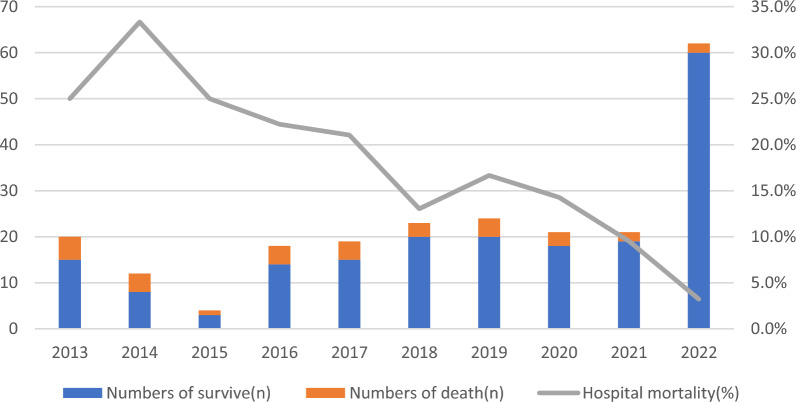
Changes in the number of surgical cases and hospital mortality rate

The present study revealed a significant decrease in the operative mortality rate over the last five years (10.6% in 2018–2022) compared to that in the previous five years (21.9% in 2013–2017, *p* = 0.023), despite the more complex procedures and lower weights in recent years (*p* < 0.05) (Table 5). Since 2019, surgical procedures have significantly decreased the annual mortality rate (Fig. 1), and a decrease in the bloodstream infection rate was also observed (11.3% in 2018–2022 vs. 23.3% in 2013–2017).

**Table 5:** Perioperative outcomes between 2013–2017 and 2018–2022

	2013–2017 (*n* = 73)	2018–2022 (*n* = 151)	*P* value
Hospital mortality, *n* (%)	16 (21.9)	16 (10.6)	0.023
Mortality of STAT1-3, *n* (%)	11 (19.0)	7 (8.5)	0.069
Mortality of STAT4-5, *n* (%)	4 (26.7)	9 (13.0)	0.186
Age at admission, days	8 (3–14)	2 (0–13)	0.005
Age at surgery, days	12 (8–20)	12 (6–19)	0.465
Weight, kg	3.5 (3.0–4.0)	3.3 (2.9–3.7)	0.008
Male, *n* (%)	53 (72.6)	98 (64.9)	0.249
Gestational age, weeks	38 (37–38)	39 (38–39)	0.000
< 37 wk *n* (%)	2 (2.7)	12 (7.9)	0.131
Prenatal diagnosis of CHD, *n* (%)	19 (26.0)	108 (71.5)	0.000
Preoperative location, *n* (%)			0.000
Pediatric ward	61 (83.6)	24 (15.9)	
NICU	8 (11.0)	12 (7.9)	
CICU	4 (5.5)	115 (76.2)	
Preoperative emergent resuscitation, *n* (%)	16 (21.9)	16 (10.6)	0.023
Emergent procedure, *n* (%)	15 (20.5)	15 (9.9)	0.029
Preoperative vasoactive support, *n* (%)	13 (17.8)	31 (20.5)	0.631
Preoperative respiratory support, *n* (%)			0.245
Room air	55 (75.3)	100 (66.2)	
Noninvasive ventilation	5 (6.8)	21 (13.9)	
Invasive mechanical ventilation	13 (17.8)	30 (19.9)	
STAT, *n* (%)			0.000
1	1 (1.4)	15 (9.9)	
2	49 (67.1)	55 (36.4)	
3	8 (11.0)	12 (7.9)	
4	15 (20.5)	68 (45.0)	
5	0 (0)	1 (0.7)	
Preoperative lac, mmol/L	2.3 (1.3–4.3)	1.5 (1.0–2.6)	0.001
Lactate on ICU arrival, mmol/L	4.0 (2.7–6.6)	2.9 (1.7–4.8)	0.007
Lac on 24 h after operation, mmol/L	3.0 (1.9–5.1)	2.2 (1.5–3.4)	0.005
Bypass, *n* (%)	69 (94.5)	141 (93.4)	0.74
Bypass time, minutes	170 (119–224)	145 (100–187)	0.134
Aortic cross‑clamp time, minutes	95 (50–125)	76 (53–106)	0.192
Circulatory arrest	5 (6.8)	25 (16.6)	0.046
Postoperative mechanical ventilation, hours	96 (66–142)	93 (47–142)	0.416
Postoperative ICU LOS, days	9 (7–13)	11 (8–16)	0.053
Postoperative hospital LOS, days	13 (10–18)	15 (11–20)	0.124
VIS	15 (9–22)	10 (7–18)	0.002
ECMO use	2 (2.7)	7 (4.6)	0.498
Delayed sternal closure	29 (39.7)	38 (25.2)	0.026
Peritoneal dialysis	14 (19.2)	31 (20.5)	0.813
Residual lesion, *n* (%)	6 (8.2)	15 (9.9)	0.680
Extubation failure, *n* (%)	10 (13.7)	13 (8.6)	0.240

Patients requiring prolonged ventilation were linked to preoperative invasive mechanical ventilation, higher VIS, and greater lactate levels 24 h after the operation, as determined by multivariate logistic regression analysis (*p* < 0.05).

Patients with prenatal diagnoses had a lower occurrence of emergent procedures and preoperative emergent resuscitation and a lower mortality rate than did those with postnatal diagnoses (*p* < 0.05) (Table 6). The most common diagnoses were TGA/IVS (*n* = 63) and TAPVC (*n* = 52). In the prenatal diagnosis group, the diagnostic rate of TGA/IVS was 21.3% (27/127), whereas it was 37.1% (36/97) in the postnatal group (*p* = 0.009). Additionally, between the prenatal diagnosis group and the postnatal diagnosis group, the diagnostic rate of TAPVC was not significantly different (28/127, 22.0% vs. 24/97, 24.7%; *p* = 0.636). Subgroup analysis of TGA/IVS patients revealed greater mortality in the postnatal diagnosis group, although the difference did not reach statistical significance (9/36, 25.0% vs. 2/27, 7.4%; *p* = 0.069). Similarly, in the TAPVC patients, we found the same result (3/24, 12.5% vs. 2/28). 7.1%; *p* = 0.541).

**Table 6:** Patient and operative characteristics based on prenatal diagnosis of CHD

	Postnatal diagnosis group (*n* = 97)	Prenatal diagnosis group (*n* = 127)	*P* value
Hospital mortality, *n* (%)	21 (21.6)	11 (8.7)	0.006
Year of surgery, 2018–2022, *n* (%)	43 (44.3)	108 (85.0)	0.000
Age at admission, days	11 (4–17)	0 (0–9)	0.000
Age at surgery, days	15 (8–21)	11 (6–16)	0.003
Weight, kg	3.6 (3.2–4.0)	3.2 (2.9–3.5)	0.000
Male, *n* (%)	65 (67.0)	86 (67.7)	0.911
Gestational age, weeks	38 (37–39)	38 (38–39)	0.091
< 37 wk *n* (%)	3 (3.1)	11 (8.7)	0.088
Preoperative location, *n* (%)			0.000
Pediatric ward	67 (69.1)	18 (14.2)	
NICU	2 (2.1)	18 (14.2)	
CICU	28 (28.9)	91 (71.7)	
Preoperative emergent resuscitation, *n* (%)	19 (19.6)	13 (10.2)	0.047
Emergent procedure, *n* (%)	19 (19.6)	11 (8.7)	0.017
Preoperative vasoactive support, *n* (%)	1.7 (1.1–2.8)	1.7 (1.1–3.2)	0.448
Postoperative ICU LOS, days	9 (6–12)	11 (8–18)	0.001
Postoperative hospital LOS, days	13 (9–17)	16 (12–21)	0.001

Mortality was also lower among patients in the CICU than among those in the pediatric ward and NICU, and both the occurrence of emergent procedures and preoperative emergent resuscitation were lower among the CICU patients (Table 7). The lactate levels and VIS scores were lower among CICU patients. Furthermore, the lactate levels (pediatric ward vs. NICU vs. CICU, 4.0 (2.6–6.5) vs. 3.7 (3.0–6.0) vs. 2.9 (1.7–4.5), *p* < 0.05) and VIS 24 h after surgery (pediatric ward vs. NICU vs. CICU, 15.0 (10–22) vs. 14.5 (10–22.0) vs. 10.0 (7–16), *p* < 0.05) were lower among the CICU patients. However, complications were not significantly different between the locations.

**Table 7:** Comparison between pediatric ward, NICU and CICU patients

Preoperative location, *n* (%)	Emergent procedure, *n* (%)				Preoperative emergent resuscitation, *n* (%)				Mortality			
	No (*n* = 194)	Yes (*n* = 30)	*χ*^2^	*P* value	No (*n* = 192)	Yes (*n* = 33)	*χ*^2^	*P* value	Death (*n* = 32)	Survival (*n* = 192)	*χ*^2^	*P* value
Pediatric Ward	68 (80.0)a	17 (20.0)a			68 (80.0)a	17 (20.0)a			16 (18.8)a	69 (81.2)a		
NICU	17 (85.0)a	3 (15.0)a	5.798	0.055	14 (70.0)a	6 (30.0)a	10.692	0.005	7 (35.0)a	13 (65.0)a	12.83	0.02
CICU	109 (91.6)b	10 (8.4)b			110 (92.4)b	9 (7.6)b			9 (7.6)b	110 (92.4)b		

(a, b), if the same letters exist, it indicates that there is no statistical significance between groups

### Follow-up

At a median follow-up of 28.0 (11.0–62.3) months, the completeness of the study was 92.7%. There were 10 (5.6%) late deaths, including 4 patients (40%) diagnosed with supracardiac TAPVC, 1 (10%) patient with supracardiac TAPVC and vertical vein stenosis, 1 (10%) patient with cardiac TAPVC, 1 (10%) patient with mixed TAPVC, and 3 (30%) patients with TGA/IVS. The median age of death was 2.5 (1.0–9.8) months. Five patients died due to postoperative anastomotic and branch pulmonary vein obstruction, 3 died due to heart failure, 1 died due to necrotizing enterocolitis, and 1 died due to obstruction of the superior vena cava.

Among 168 survivors (94.4%), reinterventions occurred in 7 (4.2%) patients, including 2 (28.6%) with supracardiac TAPVC, 1 (14.3%) with cardiac TAPVC, 2 (28.6%) with TGA/IVS, 1 (14.3%) with IAA (B), and 1 (14.3%) with COA. The median age at reoperation was 12.0 (5.0–72.0) months. The most common indications for reintervention were postoperative pulmonary vein obstruction (PVO) (*n* = 3, 42.9%), pulmonary artery branch stenosis (*n* = 1, 14.3%), supravalvular stenosis (*n* = 1, 14.3%), and aortic recoarctation (*n* = 2, 28.6%).

**Fig. 2:**
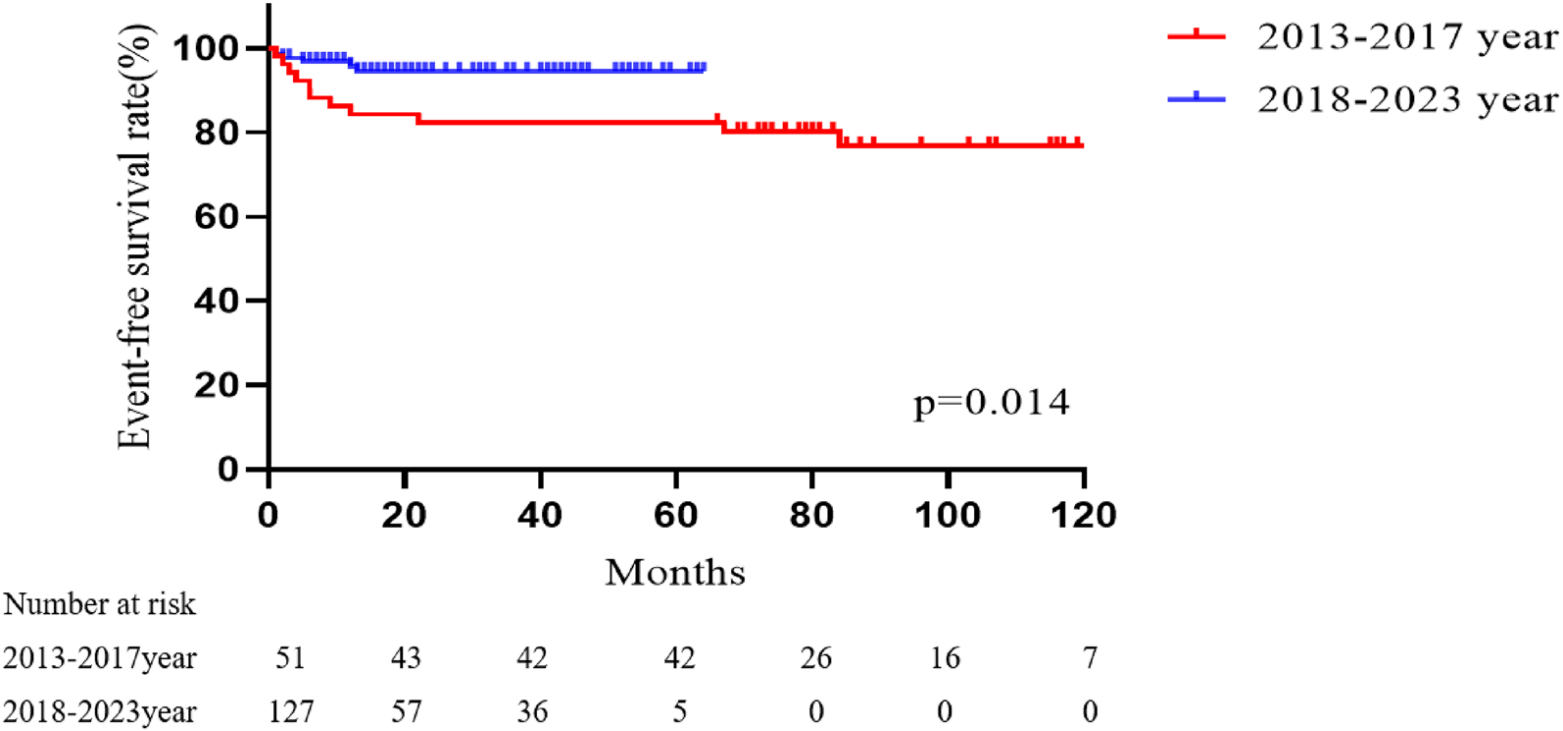
Kaplan–Meier curves comparing event-free survival rate in patients from different time periods

There were significant differences in late death and reintervention between the last five years (4.7% in 2018–2022) and the previous five years (21.6% in 2013–2017, *p* = 0.014). A comparison of survival or freedom from reintervention for the two periods is shown in Fig. 2. The surgical period (HR = 0.230, 95% CI 0.081–0.654; *p* = 0.006), prolonged ventilation (HR = 4.792, 95% CI 1.296–16.177; *p* = 0.018) and STAT category 3–5 (HR = 5.936, 95% CI 1.672–21.069; *p* = 0.006) were found to be independent risk factors for the composite end-point.

## Discussion

The mortality rate for neonatal cardiac surgery has decreased to 7.7% in some pediatric cardiac centers [7], but China's rate is still 10% [8]. As the number of neonates undergoing surgery in middle-income countries is expected to increase [9], improving surgical quality is urgent. This article includes a 10-year experience review of a single center and summarizes our treatment experience. Mortality remains a good measure of success in neonatal programs [10]. Our 10-year experience review of a single center showed an overall hospital mortality of 14.3%, and according to the Society for Thoracic Surgery Congenital Heart Surgery Database from 2014 to 2018, neonates had an operative mortality rate of 8.1% [11]. Therefore, there is still a need for indicators that could help us predict poor prognosis early. Using multivariable regression analysis, several risk factors for hospital mortality, such as gestational age, ECMO use, low birth weight, longer cardiopulmonary bypass time, STAT score, and extubation failure, have been identified [12–16]. Our findings are not consistent with those of previous cohorts.

The use of vasoactive support is common after cardiac surgery in infants, and a high VIS has been found to be significantly associated with hospital mortality, duration of mechanical ventilation, and length of ICU stay [17, 18]. VIS may also be helpful in predicting poor outcomes in newborn patients with septic shock [19]. The results of our study indicated a significant association between high VIS scores and hospital mortality, with a score greater than 15.5 at 24 h serving as an indicator to clinicians that patients are in critical condition and at high risk for PMV. Prolonged ventilation is a severe condition that poses a high risk of mortality and poor outcomes for neonates [20].

Elevated lactate levels, a marker of inadequate tissue oxygenation, have been evaluated as a biomarker to risk stratify patients undergoing cardiac surgery. In neonates following cardiac surgery, hyperlactataemia has been shown to predict a higher risk of mortality [21, 22]. In a recent study, 432 neonates underwent cardiac surgery, and a change in lactate concentration ≥ 1.6 mmol/l from cardiac ICU admission to 12 h was an independent predictor of hospital mortality [23]. Our study revealed that a lactate level of 4.8 mmol/l or higher 24 h after surgery was an independent predictor of hospital mortality. Hyperlactataemia is associated with a range of pathophysiologic states, including decreased cardiac output, inadequate oxygen delivery, and capillary leak syndrome [24], as well as a prolonged requirement for respiratory and cardiovascular support and increased mortality following cardiac surgery [25]. These findings are consistent with our findings, and our results showed that a higher level of lactate is associated with prolonged ventilation. Our findings underscore the importance of lactate and the VIS as fast, widely available biomarkers for predicting the prognosis of neonatal patients, particularly for the early detection of perfusion alterations.

During our retrospective analysis, we discovered a significant improvement in survival rates within our contemporary cohort compared to our previous study conducted between 2013 and 2017. Notably, the mortality rate in patients with critical CHD decreased as the complexity of surgery increased. This trend may reflect changes in our institutional practice patterns and other factors. We found that technical performance during neonatal cardiac surgery had a strong positive impact on survival rates, according to the findings from a study conducted by Jeffrey [26]. Additionally, several technical modifications have been made in the past five years to improve surgical outcomes, such as proactively reducing delayed sternal closure and decreasing bloodstream infection rates. To avoid arch obstruction, we used autologous vascular patches harvested from the main pulmonary artery in the reconstruction of the aortic arch for interrupted aortic arch cases. In addition, we extended the application of suture-less anastomoses to the primary repair of TAPVC.

Another significant change at our institution occurred in 2018, when there was a significant increase in the number of prenatal diagnoses for CHD compared to that in the previous period. The prognosis for surgically repaired CHD has improved dramatically in recent years, with survival rates exceeding 97% in several pediatric heart centers, including those in Beijing, Shanghai, and Guangzhou [27]. Timely diagnosis of critical CHD is key to improving outcomes, as late detection has been linked to early morbidity and mortality and higher inpatient costs [28, 29].

Our study revealed that prenatal diagnosis was associated with a decreased need for resuscitation and emergent procedures, indicating that prenatal diagnosis helps stabilize more patients before surgery. Although there was no significant difference, we found that prenatal diagnosis was beneficial for reducing the mortality of TGA/IVS and TAPVC patients. To ensure early detection, timely diagnosis, and appropriate management of CHD, Anzhen Hospital has established regularly scheduled multidisciplinary perinatal conferences to discuss prenatal diagnoses, timing and mode of delivery, and postnatal treatment programs. The benefits of prenatal diagnosis of CHD are evident in achieving these objectives.

Another change in our center was the preoperative location of the neonates. In the past 10 years, there has been a shift in the management of neonatal CHD nationally, with a trend away from the pediatric ward or NICU and towards care in the CICU. Several studies have shown that patients with certain conditions may have improved outcomes. Punkaj et al. [30] reported a study of 5376 patients and reported that the total hospital length of stay and total length of mechanical ventilation were significantly greater among NICU patients than among CICU patients, but there was no significant difference in mortality among patients undergoing cardiac surgery. Moreover, there was no subgroup analysis of newborn patients in this study. Another recent study revealed that neonatal admission to an ICU specializing in cardiac care is associated with shorter hospital and ICU LOSs, fewer days of mechanical ventilation and significantly decreased hospital costs [31]. In our study, we found that ICU and hospital stay durations, as well as the total length of mechanical ventilation, were comparable between NICU and CICU patients. There may be several reasons for this observation. First, the sample size of patients in the NICU group was small. Additionally, transfers between units may have been influenced by changes in patients’ medical conditions following their initial admission. Notably, 12% of patients from the NICU and Paediatric Ward were transferred to the ICU prior to surgery. For the independent risk factors for mortality, we found that the lactate concentration at 24 h after the operation and the VIS were lower in CICU patients than in HCs, which aligns with the mortality outcomes observed in the CICU.

Our study has revealed some interesting insights into the advantages of intensive care specialization in neonates who undergo congenital heart surgery. Previous studies have identified the importance of teamwork in providing optimal care for neonates who undergo congenital heart surgery [32]. Our study builds on this research by emphasizing the benefits of a specialized approach. By bringing together experts in cardiac medicine, cardiac surgery, intensive care, and functional examinations, we were able to improve the outcomes of our patients. This approach is especially important for neonates who have coexisting conditions or noncardiac morbidities, such as hypoglycaemia, indirect hyperbilirubinemia, cholestasis, or necrotizing enterocolitis (NEC) [33].

On the basis of our retrospective study, we found that effective neonatal cardiac surgery currently has excellent long-term outcomes. Moreover, our research revealed that TAPVC has the worst prognosis, and PVO was the main cause of reintervention, consistent with previous findings [34]. Moreover, we found that the earlier the onset of PVO was, the worse the prognosis was. Seven patients experienced postoperative PVO within 6 months after surgery, 5 ultimately died, and Seale et al. reported similar findings [35]. Therefore, close observation and early intervention during follow-up are necessary for TAPVC patients.

In summary, neonatal cardiac surgery can be performed with low mortality rates and favorable outcomes. Our study revealed that both the VIS and lactate level may be useful for predicting neonatal mortality following cardiac surgery. Specifically, a high VIS (> 15.5) and lactate level (> 4.8 mmol/l) 24 h postoperation indicate to clinicians that there is an increased risk of poor outcomes in these patients. For newborns with prenatal diagnoses, our results indicate a lower mortality rate after surgery. Additionally, the preoperative location of neonates with congenital heart disease may have an impact on surgical outcomes, and admission to the CICU prior to surgery has been shown to be beneficial. Although most patients have good long-term clinical outcomes, late death and reintervention are inevitable in some patients.

We hope that our findings will encourage other centers to adopt similar approaches and further advance the field of pediatric cardiac medicine.

### Limitations

This study is subject to certain limitations, including a retrospective study design and a small case volume. The results of this single institutional study require further exploration, due to the variation in preoperative medical management and surgical techniques utilized across the study period. And due to insufficient work experience, some clinical data were missing. The prolonged duration of the study also complicates the assessment of the effects of changes in practice over time. To derive more meaningful insights, longer-term follow-up outcomes should also be considered. Demonstrating a benefit of prenatal diagnosis in CHD on postnatal outcomes remains challenging. Hence, future studies with larger sample sizes would be invaluable. In order to fully validate our thesis, we recommend undertaking multi-center studies.

## Data Availability

All data points generated or analyzed during this study are included in this article and there are no further underlying data necessary to reproduce the results.

## References

[1] Zhao QM, Ma XJ, Ge XL (2014). Neonatal Congenital Heart Disease screening group. Pulse oximetry with clinical assessment to screen for congenital heart disease in neonates in China: a prospective study. Lancet.

[2] Tweddell JS (2016). Advances in neonatal cardiac surgery: recent advances, the low-hanging fruit, what is on the horizon and the next moonshot. Curr Opin Cardiol.

[3] Jessica H, Eric MG, William TM (2022). Perioperative metabolites are associated with adverse neonatal congenital heart disease surgical outcomes. J Am Heart Assoc.

[4] Di Nora T, Fabrizio M, Giovanni L (2015). Hepatic and renal effects of cardiopulmonary bypass. Best Pract Res Clin Anaesthesiol.

[5] Butts RJ, Scheurer MA, Zyblewski SC (2014). A composite outcome for neonatal cardiac surgery research. J Thorac Cardiovasc Surg.

[6] Gaies MG, Jeffries HE, Niebler RA (2014). Vasoactive-inotropic score is associated with outcome after infant cardiac surgery: an analysis from the pediatric cardiac critical care consortium and virtual PICU system registries. Pediatr Crit Care Med.

[7] Shuhaiber J, Gauvreau K, Thiagarjan R (2012). Congenital heart surgeon's technical proficiency affects neonatal hospital survival. J Thorac Cardiovasc Surg.

[8] Ai CC, Jia B (2016). Early intervention of neonates with critical congenital heart diseases. Chin J Thorac Cardiovasc Surg.

[9] Gunasekara CM, Moynihan K, Sudhakar A (2020). Neonatal cardiac surgery in low resource settings: implications of birth weight. Arch Dis Child.

[10] Padley JR, Cole AD, Pye VE (2011). Five-year analysis of operative mortality and neonatal outcomes in congenital heart disease. Heart Lung Circ.

[11] The STS Congenital Heart Surgery Database. Retrieved March 1, 2020; from https://www.sts.org/registries-research-center/sts-national-database/congenital-heart-surgery-database.

[12] O’Brien SM, Clarke DR, Jacobs JP (2009). An empirically based tool for analyzing mortality associated with congenital heart surgery. J Thorac Cardiovasc Surg.

[13] Kansy A, Tobota Z, Maruszewski P (2010). Analysis of 14,843 neonatal congenital heart surgical procedures in the European Association for Cardiothoracic Surgery Congenital Database. Ann Thorac Surg.

[14] Brian DB, Christopher WM, Eric MG (2017). Variation in extubation failure rates after neonatal congenital heart surgery across Pediatric Cardiac Critical Care Consortium hospitals. J Thorac Cardiovasc Surg.

[15] Fabio SN, Justin JE, Danielle G (2021). Relationship between gestational age and outcomes after congenital heart surgery. Ann Thorac Surg.

[16] Ahmed AE, Osman OA, Ragab SD (2022). Neonatal congenital heart surgery: contemporary outcomes and risk profile. J Cardiothorac Surg.

[17] Hoffman TM, Wernovsky G, Atz AM (2003). Efficacy and safety of milrinone in preventing low cardiac output syndrome in infants and children after corrective surgery for congenital heart disease. Circulation.

[18] Gaies MG, Gurney JG, Yen AH (2010). Vasoactive-inotropic score as a predictor of morbidity and mortality in infants after cardiopulmonary bypass. Pediatr Crit Care Med.

[19] Salih D, Sevilay T, Nilgun K (2022). Vasoactive inotropic score as a predictor of mortality in neonatal septic shock. J Trop Pediatr.

[20] Michaël S, Nicolas S, Krystale BG (2021). Long-term mechanical ventilation in neonates: a 10-year overview and predictive model. Front Pediatr.

[21] Cheung P, Chui N, Joffe AR (2005). Postoperative lactate concentrations predict the outcome of infants aged 6 weeks or less after intracardiac surgery: a cohort follow-up to 18 months. J Thorac Cardiovasc Surg.

[22] Schumacher KR, Reichel RA, Vlasic JR (2014). Rate of increase of serum lactate level risk-stratifies infants after surgery for congenital heart disease. J Thorac Cardiovasc Surg.

[23] Eleonore V, Steven JS, Meena N (2021). Hyperlactataemia as a predictor of adverse outcomes post-cardiac surgery in neonates with congenital heart disease. Cardiol Young.

[24] Draben L (2018). Hyperlactatemia and patient outcomes after pediatric cardiac surgery. Crit Care Nurse.

[25] O'Connor E, Fraser JF (2012). The interpretation of perioperative lactate abnormalities in patients undergoing cardiac surgery. Anaesth Intensive Care.

[26] Jeffrey S, Kimberlee G, Ravi T (2012). Congenital heart surgeon's technical proficiency affects neonatal hospital survival. J Thorac Cardiovasc Surg.

[27] Xiao-JM G-YH (2018). Current status of screening, diagnosis, and treatment of neonatal congenital heart disease in China. World J Pediatr.

[28] Oster ME, Lee KA, Honein MA (2013). Temporal trends in survival among infants with critical congenital heart defects. Pediatrics.

[29] Peterson C, Dawson A, Grosse SD (2013). Hospitalizations, costs, and mortality among infants with critical congenital heart disease: how important is timely detection?. Birth Defects Res A Clin Mol Teratol.

[30] Punkaj G, Brandon WB, Tommy RN (2014). Impact of preoperative location on outcomes in congenital heart surgery. Ann Thorac Surg.

[31] Joyce TJ, Jacob FW, Shaji CM (2018). Admission to dedicated pediatric cardiac intensive care units is associated with decreased resource use in neonatal cardiac surgery. J Thorac Cardiovasc Surg.

[32] Burstein DS, Rossi AF, Jacobs JP (2010). Variation in models of care delivery for children undergoing congenital heart surgery in the United States. World J Pediatr Congenit Heart Surg.

[33] Hamrick S, Ball MK, Rajgarhia A (2021). Integrated cardiac care models of neonates with congenital heart disease: the evolving role of the neonatologist. J Perinat Med.

[34] Erchao J, Hailong Q, Xiaobing L (2021). The outcomes of total anomalous pulmonary venous connection in neonates-10-year experience at a single center. Front Cardiovasc Med.

[35] Seale AN, Uemura H, Webber SA (2013). Total anomalous pulmonary venous connection: outcome of postoperative pulmonary venous obstruction. J Thorac Cardiovasc Surg.

